# Relationship between Frequency of Meals Comprising Staple Grain, Main, and Side Dishes and Nutritional Adequacy in Japanese Adults: A Cross-Sectional Study

**DOI:** 10.3390/nu16111628

**Published:** 2024-05-26

**Authors:** Aiko Narumi-Hyakutake, Kimika Yamada, Yatsuki Yanagihara

**Affiliations:** 1 Department of Nutrition, Faculty of Nutrition, Kobe Gakuin University, 518 Igawadani Arise, Nishi-ku, Kobe 651-2180, Japan; 2 Graduate School of Life Sciences, Showa Women’s University, 1-7-57 Taishido, Setagaya-ku, Tokyo 154-8533, Japan; 3 College of Gastronomy Management, Ritsumeikan University, 1-1-1 Nojihigashi, Kusatsu 525-8577, Japan; yanagi87@fc.ritsumei.ac.jp

**Keywords:** SMS meals, dietary intake, nutrient intake, nutritional adequacy, overall diet quality

## Abstract

Meals comprising a staple grain, a main, and side dishes (SMS meals) promote good dietary intake, yet limited studies have examined their multifactorial relationship with dietary intake. We investigated how demographic characteristics, lifestyle, and dietary habits affect the relationship between SMS meal frequency and nutrient intake adequacy. This cross-sectional study analyzed survey data from 331 Japanese adults (208 men and 123 women) aged 30–69 years in February 2019. SMS meal frequency was evaluated according to respondents’ answers to how many days a week they consumed ≥2 daily SMS meals. Dietary intake was evaluated using the brief self-administered diet history questionnaire. Differences in nutrient intake adequacy among groups according to SMS meal frequency were determined using multiple logistic regression. Less frequent SMS meals correlated with a lower intake of protein, water-soluble vitamins, and certain minerals, with more individuals falling below the estimated average requirement for nutrient intake. However, no relationship was found between SMS meal frequency and nutrient intake concerning tentative dietary goals for preventing lifestyle-related diseases. Our findings indicate that adopting SMS meals could achieve adequate intake of some nutrients and enhance overall diet quality among Japanese adults.

## 1. Introduction

A traditional Japanese diet has been recognized for supporting the health and longevity of Japanese individuals [1]. The Japanese diet is associated with low all-cause and cardiovascular mortalities [2,3,4]. A Japanese meal characteristically combines multiple dishes, exemplified by the notion of ichiju sansai—one soup and three dishes (a staple grain, main, and side dish combination, or SMS meal) [5]. The main ingredient of the staple grain dish is rice, bread, noodles, or pasta; that of the main dish is fish, meat, eggs, and/or soybean products; and that of the side dish is vegetables, mushrooms, seaweed, and/or potatoes [6]. An SMS meal may contribute to good nutritional intake due to the consumption of a variety of foods by combining several dishes. Kakutani et al. reported that the frequent consumption of SMS meals was associated with a higher intake of protein, *n*-6 and *n*-3 polyunsaturated fatty acids, total dietary fiber, potassium (K), calcium (Ca), magnesium (Mg), iron (Fe), copper (Cu), β-carotene, α-tocopherol, folate, and vitamins (K, B1, B3, B5, and C), and a lower intake of carbohydrates, including sugars, oligosaccharides, and polysaccharides, in both men and women [7]. In related research, Koyama et al. found that a higher frequency of SMS meals was linked to a higher intake of energy, Na, K, Ca, Fe, vitamin B1, vitamin B2, and vitamin C. In addition, participants who consumed fewer SMS meals were less likely to meet the Dietary Reference Intakes (DRIs) for Ca, vitamin A, vitamin B1, vitamin B2, and vitamin C [8]. Ishikawa-Tanaka et al. found that a more frequent daily intake of SMS meals was associated with a higher intake of most nutrients and food groups; moreover, participants who consumed SMS meals at least twice a day met the DRIs more consistently compared to those who consumed SMS meals once daily or less [9]. In this context, the ‘Health Japan 21′ (second term) program was developed to increase the proportion of individuals who consume two or more SMS meals daily to 80% by 2023 [10]. However, there is not enough evidence on the association between SMS meals and adequacy of nutrient intake. Ishikawa et al. investigated the relationship between SMS meals and nutrient intake using one-day dietary records. Thus, they could not assess the habitual frequency of SMS meal consumption and the associated nutrient intake [9]. Koyama et al. considered only some of the nutrients for which indicators have been established in DRIs and did not evaluate all nutrients [8]. Additionally, although the above studies examined the adequacy of individual nutrient intakes, they did not examine the overall adequacy of nutrient intakes. Therefore, considering that NCDs are a global public health issue, further research is needed to examine in detail the relationship between the frequency of SMS meals and the adequacy of habitual nutrient intake.

SMS meal frequency is associated with sex [11], age [9,11,12], body mass index [8,13], educational background [11,13], marital status [13], living arrangement [7,8], and lifestyle habits such as smoking [11,12,14], exercise [12,13], alcohol consumption [8,12], and eating speed [14]. Smokers have been found to have a higher intake of energy, total fat, saturated fatty acids, cholesterol, and alcohol, along with a lower intake of antioxidant vitamins and dietary fiber and a lower overall diet quality than non-smokers [15,16]. Individuals who regularly exercise have a higher diet quality than those who do not [17,18]. Furthermore, heavy alcohol consumption (≥2 drinks/day) is associated with low diet quality [17,18]. Dietary behaviors such as skipping breakfast is related to nutritional inadequacy [19,20,21]. Thus, when examining the relationship between SMS and dietary intake, it is crucial to consider and examine the factors influencing SMS meal frequency and dietary intake together. However, since previous studies have only examined these relationships according to demographic factors such as sex and age, the relationship between SMS meal frequency and dietary intake may not have been accurately estimated.

Therefore, in this study, our aims were twofold: first, to investigate the relationship between SMS meal frequency and participants’ demographic characteristics, lifestyle, and dietary habits; and second, to examine the association between SMS meal frequency and the adequacy of habitual nutrient intake, while considering potential confounders, among Japanese adults.

## 2. Materials and Methods

### 2.1. Survey Procedure

In this cross-sectional study, we analyzed data obtained from an online and postal survey conducted in February 2019. This study was conducted as part of a study using another online eye-tracking system. Therefore, participants filled out the questionnaire on basic demographic and lifestyle variables after providing informed consent. Since the questionnaire that assessed their dietary intake was not compatible with online surveys, participants who completed the online survey were asked to fill out and return a mailed questionnaire.

### 2.2. Participants

A total of 800 individuals, comprising Japanese men and women aged 30–69 years, stratified by sex and age group, were randomly recruited from a pool of 890,000 individuals registered with Asmark Co., Ltd. (Tokyo, Japan), a market research company, to complete online surveys. Participation was voluntary, and no incentives were provided. The data of individuals meeting the following criterial were excluded: (1) those who declined participation (*n* = 218); (2) those who did not complete the entire survey (*n* = 242); (3) those who did not provide essential data for analysis (*n* = 2); (4) those suspected of significantly under- or over-reporting data, corresponding to an energy intake <0.5 times the estimated energy requirement (EER) for physical activity level I (*n* = 0) or >1.5 times the EER for physical activity level III (*n* = 0); (5) those following a controlled diet under expert guidance (*n* = 6); and (6) those who were lactating (*n* = 1). Eventually, the data of 331 respondents were included in the analysis.

### 2.3. Ethical Considerations

This study was conducted according to the guidelines outlined in the Declaration of Helsinki. All procedures involving human participants were approved by the Kobe Gakuin University Ethics Review Committee (approval number: HEB19-14). Written (online) informed consent was obtained from all participants.

### 2.4. Measured Variables

#### 2.4.1. SMS Meal Frequency

Participants were asked to respond to the question, ‘How many days a week do you eat meals that combine a staple grain dish (rice, bread, or noodles), main dish (dishes with meat, fish, eggs, and/or soybean products as the main ingredient), and side dishes (small bowls/plates of vegetables, mushrooms, and/or seaweed) at least twice a day?’ [22]. Responses were scored on a 5-point scale as follows: ‘almost every day’, ‘4–5 days a week’, ‘2–3 days a week’, ‘once a week’, and ‘never’.

#### 2.4.2. Dietary Intake by Food Group and Nutrient

Dietary intake in the most recent month was evaluated by the brief-type self-administered diet history questionnaire (BDHQ), a structured questionnaire with a detailed intake calculation methodology and validity that have been previously reported [23,24]. The BDHQ includes questions on the frequency of food intake but not portion size, related eating behaviors (e.g., eating speed), and the usual cooking methods (e.g., frequency of adding soy souse). Food and beverage items contained in the BDHQ were mainly from a food list used in the National Health and Nutrition Survey of Japan, while standard portion sizes and the sizes of bowls for rice and cups for miso soup were derived from several recipe books for Japanese dishes [24]. The estimated daily intake of 58 foods, nutrients, and energy was evaluated using an ad hoc computer algorithm for the BDHQ based on the ‘Standard Tables of Food Composition in Japan’ [25].

The intake of fat, saturated fats, carbohydrates, and protein was calculated as a percentage of daily energy intake using the reported values (crude). Additionally, food intake values were energy-adjusted using the density method (i.e., the percentage of energy for energy-providing nutrients and their amounts per 4184 kJ for food groups and other nutrients) to minimize the influence of dietary misreporting.

#### 2.4.3. Determination of Habitual Nutritional Adequacy

The intake adequacy of each nutrient was determined by comparing the nutrient level with its corresponding reference value in the DRIs using a cut-point method, as previously reported [26,27]. The DRIs have different types of reference value. The estimated average requirement (EAR) aims to prevent insufficient nutrient intake, whereas the tentative dietary goal (DG), either an upper or lower limit of the intake value, aims to prevent lifestyle-related/non-communicable diseases.

The reported dietary intakes were adjusted using the EER to minimize under- or over-reporting errors inherent in a self-estimated dietary assessment. The value of EER was calculated based on age according to the Japanese DRIs using the modest physical activity category. Next, they were compared with the 2015 DRIs [28], as described in previous studies [26,27,29]. Adjustments were made using the following equation:

energy-adjusted intake (units/d) = observed intake (units/d) × EER (kJ/d)/observed energy intake (kJ/d)

An intake level below the EAR was considered inadequate. The cut-point method was used for the following 14 nutrients: protein; vitamin A (expressed as retinol activity equivalents); niacin (expressed as niacin equivalents); folate; vitamins B1, B2, B6, B12, and C; and the minerals Ca, Mg, Fe, Zn, and Cu. The niacin equivalent was calculated as niacin (mg) + protein (mg)/6000. In this study, the EAR of Fe for menstruating women was used as the EAR of Fe for all women aged 30–64 years. The intake levels falling outside the DG range for the following seven nutrients were considered as not meeting the standard DG: percentage energy (%E) from proteins, total fat, carbohydrates, total dietary fiber, sodium (Na, expressed as salt-equivalent), and K. Salt-equivalent was calculated as follows: salt (g) = 58.5/23 × Na (g). Although the EAR values for biotin, chromium, molybdenum, selenium, and iodine are available in the DRIs, these nutrients were excluded from this study because of incomplete data in Japan’s food composition tables.

#### 2.4.4. Other Variables

Sex, age, height, weight, and frequency of weekly alcohol consumption (>5 days, 1–4 days, <1 day, or teetotal) were obtained from the BDHQ. Body mass index was calculated by dividing weight by height squared. Other parameters included educational background (graduated from junior high school, high school/vocational school, technical college/junior college, university, or above), annual household income (<3,000,000 yen, 3,000,000–6,000,000 yen, 6,000,000–9,000,000 yen, ≥9,000,000 yen), marital status (married or separated, divorced, widowed, or unmarried), currently smoking (yes or no), daily walking duration (>1 h, 30 min–1 h, <30 min, or rare), average daily sleep duration (>7 h, 6–7 h, <6 h), skipping breakfast (yes or no), and use of food labels when purchasing food (yes or no).

### 2.5. Statistical Analysis

A relatively low proportion of participants reported SMS meal frequency of ‘once a week’ (23(6.9%)) or ‘never’ (37(11.2%)); therefore, participants were categorized into four groups: those who had two or more SMS meals ‘almost every day of the week’, ‘4–5 days a week’, ‘2–3 days a week’, and ‘once a week or never’. The differences in characteristics between the groups stratified by SMS meal frequency were compared using the chi-square and Kruskal–Wallis tests for categorical and continuous variables, respectively. Multiple comparisons were further conducted using the post-hoc analysis corrected by Bonferroni adjustment. The between-group differences in the mean nutrient and food intake were compared using a covariate analysis (ANCOVA). Potential confounders considered in the multivariate model were sex, age (years, continuous), marital status, skipping breakfast, and food label use, which were all significantly different (*p* < 0.05) between groups stratified by SMS meal frequency. In addition, body mass index (kg/m^2^, continuous), smoking status, daily walking duration, and frequency of weekly alcohol consumption were also included in the multivariate model as confounders, as these were related to dietary intake. Linear regression analysis was used to test the trend of nutrient and food intakes across the four groups. The correlation between energy-adjusted nutrients and food intake was assessed using Spearman’s rank correlation coefficient.

The proportion of participants with a nutrient intake below the EAR or falling outside the DG range was calculated. The between-group differences in the proportion of participants ‘not meeting the DRIs’ were assessed using logistic regression analysis after adjusting for potential confounders. The *p*-value for the linear trend of prevalence of nutritional inadequacy between the groups was estimated by modelling the frequency categories as a continuous variable.

The number of nutrients that did not meet each EAR value and DG range were counted to examine the overall nutritional adequacy of each participant’s dietary intake. The between-group differences in the number of nutrients ‘not meeting the DRIs’ were assessed using multiple regression analysis. The aforementioned potential confounders were considered in this model as well. Linear regression analysis was used to test the trend of the number of nutrients not meeting dietary reference intakes across the groups.

All statistical analyses were performed using IBM SPSS Statistics for Mac OS, version 25.0 (IBM Corp., Armonk, NY, USA). All reported *p*-values were two-tailed; a *p*-value < 0.05 was considered statistically significant.

## 3. Results

### 3.1. Participant Characteristics

Participant characteristics are listed in Table 1. Overall, 62.8% of participants were men, and the average age was 48.8 ± 10.2 years. Regarding SMS meal frequency, 132 participants (39.9%) responded that they had two or more SMS meals ‘almost every day’ of the week, whilst 65 (19.6%), 74 (22.4%), and 60 (18.1%) responded ‘4–5 days a week’, ‘2–3 days a week’, and ‘once a week or never’, respectively. Those who reported that they consumed SMS meals ‘almost every day’ were primarily women, older individuals, and married individuals. In addition, most of these participants did not skip breakfast or check food labels when purchasing food. No relationship was observed between SMS meal frequency and body mass index, educational background, annual household income, smoking status, daily walking duration, or alcohol consumption.

### 3.2. SMS Meals and Habitual Nutrient Intakes

Table S1 presents the energy and nutrient intakes stratified by the frequency of days per week that participants consumed two or more SMS meals. In the crude model, significant between-group differences were observed in the intake of all nutrients except dietary fiber, protein, vitamin A, niacin equivalent, Mg, Zn, and Cu. Trend-level between-group differences were observed for all nutrients except protein, niacin equivalent, Zn, and Cu. In the multivariate model, significant between-group differences were observed in the intake of protein, Zn, and vitamins B1, B6, and B12. A lower intake of protein (%E, g/d); K; niacin equivalent; vitamins B1, B2, B6, and B12; folate; Fe; and Zn was associated with lower SMS meal frequency. No relationship was observed between the intake of other nutrients and SMS meal frequency.

### 3.3. SMS Meals and Adequacy of Nutrient Intake

Table 2 presents the proportion of participants who did not meet the DRIs, stratified by weekly frequency of SMS meals and odds ratio (OR) in participants who consumed two or more SMS meals ‘almost every day’ of the week. When DG was used as an indicator, most participants’ dietary fiber and salt intake deviated from the DG range (86.1% and 98.5%, respectively). The proportion of participants with protein intake falling outside the DG range was higher among those who consumed two or more SMS meals (‘2–3 days a week’ and ‘once a week or never’) than among those who consumed SMS meals ‘almost every day’, with a high OR and trend-level association. When EAR was used as an indicator, only a few participants had protein, vitamin B12, and Cu intakes below the EAR (0.9%, 0.6%, and 0.6%, respectively). In addition, none of participants had niacin equivalent intake below the EAR. The proportion of participants with vitamins B2, B6, and C intakes below the EAR was higher among those who consumed two or more SMS meals ‘once a week or never’ than among those who consumed SMS meals ‘almost every day’, with a high OR and trend-level association. The proportion of participants with folate intake below the EAR increased as SMS meal frequency decreased.

Table 3 presents the adequacy of overall nutrient intake stratified by SMS meal frequency. The mean number of nutrients with intake below the EAR was 2.0 (standard error [SE]: 0.2), 2.1 (SE: 0.3), 2.7 (SE: 0.3), and 3.1 (SE: 0.3) among participants who consumed two or more SMS meals ‘almost every day’, ‘4–5 days a week’, ‘2–3 days a week’, and ‘once a week or never’, respectively. The results exhibited an increase with the decrease of SMS meal frequency (*p* trend = 0.002). The mean number of nutrients with an intake falling outside the DG range was 4.1 (SE: 0.1), 3.8 (SE: 0.2), 4.2 (SE: 0.2), and 4.2 (SE: 0.2) in participants who consumed two or more SMS meals ‘almost every day’, ‘4–5 days a week’, ‘2–3 days a week’, and ‘once a week or never’, respectively, with no significant between-group differences.

### 3.4. SMS Meals and Food Group Intakes

Table S2 presents the dietary intake according to food group stratified by SMS meal frequency. In the crude model, between-group differences and trends were observed in the intake of most food groups. In contrast, in the multivariate model, between-group differences were observed only for the intake of noodles and seafood. In the multivariate model, a lower SMS meal frequency was associated with a higher intake of noodles, sugars and confections, and soft drinks, and a lower intake of seaweed, seafood, and eggs.

## 4. Discussion

To our knowledge, this is the first study to examine the relationship between SMS meal frequency and adequacy of nutrient intake and its potential confounders. Our analysis revealed that SMS meal frequency was associated with the dietary intake of several nutrients, food groups, and nutritional adequacy. Nevertheless, dietary fiber and salt intakes frequently deviated from the recommended range, even among individuals who consumed SMS meals frequently, and this occurred independently of SMS meal frequency. Furthermore, no relationship was found between SMS meal frequency and nutrient intake adequacy in preventing lifestyle-related diseases.

In our study, the proportion of respondents reporting consumption of ≥2 SMS meals almost daily during the study week (39.9%) was similar to that observed in a previous Japanese survey (40.6%). Conversely, the proportion of participants who rarely consumed SMS meals in our study was 18.1%, which was higher than that reported in the previous survey (11.9%) [22]. This difference may be due to sampling bias in this study, such as a greater number of male participants and those who were relatively young. Alternatively, it may be due to the influence of other factors (e.g., food consciousness) that were not investigated in this study.

Sex-related differences in the quality and quantity of foods and nutrition have been reported in previous work [30]. In our study, women ate SMS meals more often than men, which supports previous reports [7,10,11,12]. Wardle et al. discovered in a multi-national study that women place greater importance on healthy eating than men and that health beliefs explain a large proportion of dietary behavior [31]. A survey of Japanese adults found that women prioritized health when choosing foods and had better nutrition-related knowledge than men [32], which suggests that such sex differences in awareness and attitudes may also explain the difference in SMS meal frequency. Individuals who consumed more frequent SMS meals were older than those who consumed infrequent SMS meals. The Conscious Survey for Shokuiku 2023 reported that the percentage of individuals who consumed at least two daily SMS meals almost daily increased with age for both sexes [22]. Similar findings have been reported by Ishikawa-Tanaka et al. and Sakurai et al. [9,12]. On the other hand, other studies have reported no relationship between SMS meal frequency and age [7,8,33]. However, these studies had limitations, such as being limited to a specific age group (over 75 years old [33]) or participant characteristics (university and vocational school students [7], factory workers [8]). Thus, the differences in age range and characteristics between study participants may explain some of the differences in results. We observed that married individuals consumed two or more SMS meals almost daily, exceeding the frequency among unmarried individuals. Several studies have reported that marital status has an effect on health [34,35,36]. While these effects have been partially attributed to the characteristics of individuals who choose to marry, married individuals may have greater social support, a higher standard of living, and a healthier lifestyle than unmarried individuals [34,36,37]. These tendencies may be reflected in the trends concerning SMS meal frequency.

Our study revealed that individuals who consumed two or more SMS meals almost every day rarely skipped breakfast. Skipping breakfast reduces the number of daily meals, which inevitably reduces SMS meal frequency. Women and older individuals skip breakfast less often than men and younger individuals [38,39]. This trend was observed in the study participants with a high SMS meal frequency, which may have resulted in significant between-group differences. In our study, individuals who consumed two or more SMS meals almost daily were more likely to check food labels when purchasing food. This may be explained by a greater level of health awareness in individuals who tend to check food labels. Indeed, according to one systematic review, individuals who check food labels are keenly interested in health and are aware of food-related guidelines [40].

In our study, SMS meal frequency was associated with the intake of several nutrients. Previous studies have reported that SMS meal frequency is associated with the intake of more nutrients. For example, Ishikawa-Tanaka et al. reported that participants who consumed SMS meals at least twice a day met the reference values for almost all nutrients specified in the DRIs, compared with those who consumed SMS meals once a day or less [9]. Kakutani et al. and Koyama et al. reported similar findings [7,8]. However, these previous studies did not take into account the lifestyle and eating habits that affect SMS meal frequency and food intake. In our study, the results of the crude model were similar to those in the abovementioned studies; therefore, prior research may have overestimated the relationship between SMS meal frequency and dietary intake. Even after adjusting for lifestyle and dietary habits in our study, SMS meal frequency was correlated with the number of nutrients below the EAR. An SMS meal indicates that more dishes and foods are consumed per meal. Takabayashi et al. reported that the number and variety of dishes consumed by the general Japanese adult population contributes positively to nutrient intake [41]. Therefore, frequent SMS meals may effectively prevent inadequate nutrient intake. We also found that dietary fiber and salt intakes were not within the DG range in many participants and that the intakes of these nutrients were unrelated to SMS meal frequency. Previous studies have reported that the proportion of individuals with a dietary fiber intake outside the DG range was low among those with a high SMS meal frequency. However, a dietary fiber intake falling outside the range of DG was observed in nearly 50% of individuals, even among those with the highest SMS meal frequency. Moreover, the proportion of excessive salt intake was >95% among all SMS meals groups and this increased with the frequency of SMS meals [9]. Another study, which included a nationwide sample in Japan, found that the proportion of younger individuals with a dietary fiber intake below the DG range was higher than that among other age groups, and this consisted of 44.9–64.9% men and 30.9–72.2% women. Furthermore, the percentage of adults whose salt intake exceeded the DG range was higher in the older age group, comprising 90.8–94.6% of men and 88.7–98.9% of women [42]. In summary, dietary fiber and salt intake remain significant nutritional concerns among Japanese individuals, irrespective of SMS meal consumption.

In this study, participants who consumed SMS meals less frequently had less seaweed, seafood, and egg intake. Considering the definition of SMS meals used in this study, these foods were likely to have been included in their main or side dishes. Furthermore, a positive correlation was observed between the intakes of seaweed, seafood, and eggs and that of proteins, potassium, B vitamins, vitamin C, and Fe (data not presented); therefore, SMS meals may have contributed to adequate nutritional status owing to the intake of these foods.

Participants who reported consuming two or more SMS meals on fewer days reported a high intake of noodles and pasta, sugars and confections, and soft drinks. The noodles-and-pasta category of the BDHQ survey used in this study comprised udon, hiyamugi, somen, ramen, instant noodles, spaghetti, and macaroni [23]. In Japan, pasta and noodles are often consumed as a single dish with various other foods such as vegetables, eggs, and meats. Therefore, selecting noodles or pasta as the staple food may not lead to the formation of SMS meals. Furthermore, participants who consumed a meal where noodles or pasta served as the staple grain and sole dish may not have interpreted that as an SMS meal, even though it contained enough ingredients to be considered an SMS meal including a staple grain, main, and side dish. We observed a higher intake of sugars, confections, and soft drinks in participants with a lower SMS meal frequency. Participants who consumed frequent SMS meals were seemingly more interested in health—they were less likely to skip breakfast and more likely to check food labels. Therefore, these individuals may be more likely to actively avoid sweets and confections. Furthermore, the choice of SMS meals might inherently lead individuals to refrain from foods such as sweets and confections; however, drawing a definitive conclusion solely from this study is not possible, and further investigations are warranted.

This study has some limitations that should be noted. Firstly, participants were selected from among individuals registered at an online surveying company. In addition, since the daily and dietary habits were surveyed online, these might have been over-represented given the presence of participants with a high internet literacy. Therefore, the groups may not adequately represent the general population. However, our study participants were stratified by sex and age and were randomly selected from among 890,000 individuals. Their SMS meal frequency distribution results did not diverge from the results of a previous survey in Japan [22]. Therefore, they can be considered a fair sample of the general Japanese adult population for investigating the relationship between SMS meals and dietary intake. Second, the distribution of the BDHQ by mail after evaluating the online survey responses resulted in a time lag between both surveys. However, because both surveys were conducted in February 2019, it is unlikely that the participants’ dietary habits and intake would have considerably changed between the two surveys. Seasonal dietary changes were negligible and thus may have had little impact on the study results. Third, because this study evaluated SMS meal frequency based on subjective reporting, the validity of the assessment is open to debate. However, a previous study reported no major differences in the results [9], thus suggesting no critical defects in the assessment method used. Fourth, this study used the BDHQ to assess meals; hence, the exact foods comprising the staple grain, main, and side dishes the participants consumed could not be identified. Therefore, studies that use a diet recording method over several days or a 24 h recall method are needed to investigate the relationship between SMS meal frequency and nutrient intake further. Fifth, we investigated the known factors affecting SMS meals and dietary intake, yet the effects of other potential confounding factors were not investigated. Sixth, this study’s cross-sectional nature makes it difficult to attribute any causal associations. Therefore, longitudinal studies are needed to investigate the causal association between SMS meal frequency and nutrient intake in the future.

## 5. Conclusions

We found that SMS meal frequency is associated with the dietary intake of several nutrients, food groups, and nutritional adequacy; these insights may help to guide interventions that aim to prevent nutrient deficits, such as interventions to prevent undernutrition in older adults and to address thinness in young women. Conversely, it is necessary to identify the factors that are barriers to SMS meals among groups who have a low frequency of SMS diet, such as men and the younger generation. In addition, dietary fiber and salt intake are challenging issues that were found to be independent of SMS meal frequency. To solve these problems and use SMS meals to prevent lifestyle diseases, it is necessary to further investigate the appropriate choice of ingredients for each dish in SMS meals. Moreover, longitudinal studies are needed to determine whether SMS diets improve the adequacy of nutrient intake and health status.

## Figures and Tables

**Table 1:** Participant characteristics stratified by SMS meal * frequency.

	Total (*n* = 331)		Almost Every Day (*n* = 132, 39.9%)		4–5 Days/Week (*n* = 65, 19.6%)		2–3 Days/Week (*n* = 74, 22.4%)		Once a Week or Never (*n* = 60, 18.1%)		*p*
Sex, *n*, (%)											0.03
Men	208	(62.8)	72	(34.6)	43	(20.7)	47	(22.6)	46	(22.1)	
Women	123	(37.2)	60	(48.8)	22	(17.9)	27	(22.0)	14	(11.4)	
Age (years), mean, SD	48.8	10.2	51.5	10.2	47.6	8.9	47.3	10.8	46.1	9.9	<0.01
BMI † (kg/m^2^), mean, SD	22.3	3.4	21.9	3.4	22.6	3.7	22.7	3.5	22.4	3.3	0.57
Educational background, *n*, (%)											
High school or junior high school	50	(15.1)	23	(46.0)	5	(10.0)	12	(24.0)	10	(20.0)	0.54
Junior college or vocational technical school	58	(17.5)	26	(44.8)	10	(17.2)	13	(22.4)	9	(15.5)	
University or higher	223	(67.4)	83	(37.2)	50	(22.4)	49	(22.0)	41	(18.4)	
Annual household income, *n*, (%)											0.10
<3,000,000 yen	44	(13.3)	16	(36.4)	6	(13.6)	11	(25.0)	11	(25.0)	
3,000,000–6,000,000 yen	105	(31.7)	38	(36.2)	16	(15.2)	23	(21.9)	28	(26.7)	
6,000,000–9,000,000 yen	87	(26.3)	36	(41.4)	21	(24.1)	18	(20.7)	12	(13.8)	
>9,000,000 yen	95	(28.7)	42	(44.2)	22	(23.2)	22	(23.2)	9	(9.5)	
Marital status											<0.01
Married	88	(26.6)	28	(31.8)	16	(18.2)	17	(19.3)	27	(30.7)	
Unmarried	243	(73.4)	104	(42.8)	49	(20.2)	57	(23.5)	33	(13.6)	
Currently smoking, *n*, (%)											0.62
No	268	(81.0)	109	(40.7)	54	(20.1)	56	(20.9)	49	(18.3)	
Yes	63	(19.0)	23	(36.5)	11	(17.5)	18	(28.6)	11	(17.5)	
Walking duration/day, *n*, (%)											0.25
>1 h	74	(22.4)	39	(52.7)	10	(13.5)	16	(21.6)	9	(12.2)	
30 min–1 h	127	(38.4)	47	(37.0)	27	(21.3)	29	(22.8)	24	(18.9)	
<30 min or rare	130	(39.3)	46	(35.4)	28	(21.5)	29	(22.3)	27	(20.8)	
Sleep duration/day, *n*, (%)											0.12
>7 h	138	(41.7)	56	(40.6)	25	(18.1)	31	(22.5)	26	(18.8)	
6–7 h	130	(39.3)	60	(46.2)	25	(19.2)	28	(21.5)	17	(13.1)	
<6 h	63	(19.0)	16	(25.4)	15	(23.8)	15	(23.8)	17	(27.0)	
Frequency of alcohol consumption/week, *n*, (%)											0.37
>5 days	92	(27.8)	41	(44.6)	20	(21.7)	17	(18.5)	14	(15.2)	
1–4 days	94	(28.4)	29	(30.9)	22	(23.4)	23	(24.5)	20	(21.3)	
<1 day or teetotal	145	(43.8)	62	(42.8)	23	(15.9)	34	(23.4)	26	(17.9)	
Skipping breakfast, *n*, (%)											<0.01
No	249	(75.2)	118	(47.4)	47	(18.9)	53	(21.3)	31	(12.4)	
Yes	82	(24.8)	14	(17.1)	18	(22.0)	21	(25.6)	29	(35.4)	
Food label use, *n*, (%)											<0.01
Yes	174	(52.6)	85	(48.9)	34	(19.5)	33	(19.0)	22	(12.6)	
No	157	(47.4)	47	(29.9)	31	(19.7)	41	(26.1)	38	(24.2)	

SD, standard deviation; BMI, body mass index. * An SMS meal is a meal that includes a staple, main, and side dishes together in one meal. † *p* values indicate the results for the chi-square test for categorical variables and the Kruskal–Wallis test for continuous variables.

**Table 2:** Prevalence of not meeting an estimated average requirement or tentative dietary goal of the Dietary Reference Intakes among participants (*n* = 331) categorized into four groups based on SMS meal * frequency.

	Total	Almost Every Day (*n* = 132)	4–5 Days/Week (*n* = 65)				2–3 Days/Week (*n* = 74)				Once a Week or Never (*n* = 60)				*p* Trend ¶
	Prevalence of Inadequacy (%) §	Prevalence of Inadequacy (%) §	Prevalence of Inadequacy (%) §	OR (95% CI)			Prevalence of Inadequacy (%) §	OR (95% CI) ||			Prevalence of Inadequacy (%) §	OR (95% CI)			
Nutrients with DG															
Fat	46.5	47.0	38.5	0.80	(0.42, 1.53)		47.3	1.13	(0.61, 2.11)		53.3	1.70	(0.83, 3.47)		0.16
Saturated fat	54.1	63.6	47.7	0.61	(0.32, 1.18)		50.0	0.63	(0.34, 1.18)		45.0	0.76	(0.37, 1.57)		0.30
Protein	40.2	31.8	33.8	1.03	(0.51, 2.11)		47.3	2.09	(1.07, 4.07)		56.7	2.40	(1.12, 5.14)		<0.01
Carbohydrate	48.3	52.3	43.1	0.67	(0.34, 1.31)		51.4	0.99	(0.52, 1.88)		41.7	0.69	(0.33, 1.43)		0.51
Dietary fiber	86.1	85.6	84.6	0.61	(0.24, 1.53)		90.5	1.14	(0.43, 3.02)		83.3	0.46	(0.17, 1.23)		0.32
Sodium (salt-equivalent)	98.5	100.0	98.5	-	-		97.3	-			96.7	-			0.11
Potassium	34.7	25.8	30.8	0.94	(0.46, 1.94)		41.9	1.59	(0.81, 3.11)		50.0	1.66	(0.78, 3.53)		0.10
Nutrients with EAR															
Protein	0.9	0.0	0.0	-	-		2.7	-	-		1.7	-			0.97
Vitamin A †	29.0	21.2	27.7	1.14	(0.55, 2.39)		32.4	1.38	(0.69, 2.79)		43.3	1.62	(0.76, 3.49)		0.18
Vitamin B1	65.0	56.1	63.1	0.85	(0.42, 1.71)		73.0	1.79	(0.89, 3.59)		76.7	1.55	(0.68, 3.55)		0.11
Vitamin B2	11.8	5.3	6.2	0.98	(0.25, 3.82)		16.2	2.70	(0.88, 8.27)		26.7	3.16	(1.02, 9.80)		0.02
Niacin ‡	0.0	0.0	0.0	-	-		0.0	-	-		0.0	-			-
Vitamin B6	8.2	2.3	3.1	1.54	(0.23, 10.40)		8.1	4.19	(0.93, 18.91)		26.7	14.21	(3.27, 61.64)		<0.01
Vitamin B12	0.6	0.0	1.5	-	-		1.4	-	-	-	0.0	-	-		0.92
Folate	3.0	0.0	0.0	-	-		4.1	-	-	-	11.7	-	-		0.02
Vitamin C	23.9	16.7	16.9	0.83	(0.35, 1.97)		27.0	1.42	(0.65, 3.09)		43.3	2.29	(1.00, 5.21)		0.03
Calcium	37.2	28.0	38.5	1.33	(0.67, 2.63)		41.9	1.45	(0.75, 2.80)		50.0	1.62	(0.78, 3.38)		0.16
Magnesium	26.3	18.9	21.5	0.90	(0.40, 2.01)		31.1	1.35	(0.65, 2.83)		41.7	1.44	(0.65, 3.20)		0.26
Iron	23.0	20.5	21.5	1.05	(0.42, 2.64)		27.0	1.43	(0.62, 3.33)		25.0	1.78	(0.67, 4.72)		0.20
Zinc	9.4	5.3	6.2	1.95	(0.42, 9.12)		10.8	2.35	(0.59, 9.29)		20.0	3.25	(0.82, 12.80)		0.09
Copper	0.6	0.0	0.0	-	-		1.4	-	-	-	1.7	-	-	-	1.00

OR, odds ratio; SD, standard deviation; 95% CI, 95% Confidence Interval; EAR, estimated average requirement; DG, dietary goal. * An SMS meal is a meal that includes a staple, main, and side dishes together in one meal. † Sum of retinol, β-carotene/12, α-carotene/24, and cryptoxanthin/24. ‡ Sum of niacin and protein/6000. § Adjusted for reporting error as follows: energy-adjusted intake (units/day) = observed intake (units/day) × EER (kJ/day)/observed energy intake (kJ/day). The value of EER is calculated for age and according to the Japanese DRI for modest physical activity. Percentage of participants whose nutrient intake did not meet DG or EAR of DRIs. The cut-point method compared each nutrient intake with each DRI value. || Multivariate adjusted ORs of nutrient intake inadequacy between the groups stratified by SMS meal frequency are calculated by adjusting for gender (men or women), age (years, continuous), body mass index (kg/m^2^, continuous), marital status (married, separated, divorced, widowed, or unmarried), skipping breakfast (yes or no), food label use (yes or no), currently smoking (yes or no), walking duration/day (>1 h, 30 min–1 h, <30 min, or rare), frequency of alcohol consumption/week (>5 days, 1–4 days, <1 day, or teetotal). ¶ Tests of linear trends across the four groups are calculated by treating the frequency categories as a continuous variable. Each group is assigned a score: almost every day = 1, 4–5 days/week = 2, 2–3 days/week = 3, and once a week or never = 4.

**Table 3:** Number of nutrients not meeting tentative dietary goals of the Japanese Dietary Reference Intakes and estimated average requirements status among participants stratified by SMS meal * frequency.

	Almost Every Day (*n* = 132)		4–5 Days/Week (*n* = 65)		2–3 Days/Week (*n* = 74)		Once a Week or Never (*n* = 60)		*p* †	*p* Trend ‡
	Mean	SE	Mean	SE	Mean	SE	Mean	SE		
Not meeting DG	4.1	0.1	3.8	0.2	4.2	0.2	4.2	0.2	0.17	0.50
Not meeting EAR	2.0	0.2	2.1	0.3	2.7	0.3	3.1	0.3	0.01	<0.01

SE, standard error; DG, dietary goal; EAR, estimated average requirement. * An SMS meal is a meal that includes a staple, main, and side dishes together in one meal. † *p* values are shown for covariate analysis to analyze differences among participants stratified by SMS meal adjusted for confounding variables of gender, grade (freshman, sophomore, junior, or senior), body mass index (kg/m^2^, continuous), physical activity score (0–3), living status (living alone, or living with their family or living in a dormitory), money that can be used freely in a month (less than 20,000 yen, 20,000–30,000 yen, 30,000–50,000 yen, or 50,000 yen or more). ‡ Linear regression analysis was used to test the trend of the number of nutrients not meeting dietary reference intakes across the groups.

## Data Availability

The data in this study are available upon request from the corresponding author. The data are not publicly available due to confidentiality reasons.

## Supplementary Materials

The following supporting information can be downloaded at: https://www.mdpi.com/article/10.3390/nu16111628/s1, Table S1: Habitual nutrient intakes per day among participants (n = 331) categorized into four groups based on the SMS meal * frequency; Table S2: Habitual daily food group intakes (g/4184 kJ) among participants (n = 331) categorized into four groups by SMS meal frequency.
