# Supplementary material for: Relationship between Frequency of Meals Comprising Staple Grain, Main, and Side Dishes and Nutritional Adequacy in Japanese Adults: A Cross-Sectional Study
Source: Nutrients. 2024 May 26;16(11):1628. doi: 10.3390/nu16111628 (PMC11174530; doi:10.3390/nu16111628)
Supplement: Supplementary file 1 [file nutrients-16-01628-s001.zip › nutrients-2965013-supplementary.pdf]

Table S1. Habitual nutrient intakes per day among participants (n = 331) categorized into four groups based on the SMS meal* frequency

				Crude Model										Multivariate Model§							
		Total		Almost every day (n=132)		4–5 days/week (n=65)		2–3 days/week (n=74)		Once a week or never (n=60)				Almost every day (n=132)		4–5 days/week (n=65)		2–3 days/week (n=74)		Once a week or never (n=60)			
Nutrients	unit	Mean	SE	Mean	SE	Mean	SE	Mean	SE	Mean	SE	P∣∣	P trend¶	Mean	SE	Mean	SE	Mean	SE	Mean	SE	P**	P trend¶
Energy	kJ	7578	135	8031	188	8084	253	7344	359	6322	266	-	-	7923	197	8013	269	7452	252	6503	295	-	-
Fat	% Energy	27.2	0.4	28.6	0.5	27.0	0.7	26.4	0.8	25.3	0.9	<0.01	<0.01	28.1	0.5	27.1	0.7	26.3	0.7	26.5	0.8	0.21	0.050
Saturated fat	% Energy	7.2	0.1	7.6	0.2	7.3	0.2	7.1	0.3	6.6	0.3	0.02	<0.01	7.4	0.2	7.3	0.2	7.1	0.2	6.9	0.3	0.33	0.07
Protein	% Energy	14.7	0.2	15.4	0.2	14.7	0.3	14.4	0.4	13.5	0.4	<0.01	<0.01	15.2	0.2	14.8	0.3	14.4	0.3	14.0	0.4	0.06	0.01
Carbohydrate	% Energy	50.9	0.5	49.6	0.8	50.7	1.1	51.6	1.1	53.4	1.4	0.03	<0.01	50.0	0.8	51.2	1.0	51.4	1.0	52.0	1.1	0.49	0.14
Dietary fiber	g/4184 kJ	14.8	0.2	15.2	0.4	15.3	0.5	14.2	0.4	14.0	0.6	0.23	0.04	14.8	0.4	15.4	0.5	14.4	0.5	14.4	0.6	0.49	0.37
Sodium (salt-equivalent)	g/4184 kJ	13.4	0.2	12.8	0.2	13.2	0.4	13.8	0.4	14.4	0.5	0.01	<0.01	13.3	0.2	13.2	0.3	13.7	0.3	13.7	0.4	0.54	0.23
Potassium	mg/4184 kJ	3197	47	3347	71	3267	91	3036	96	2986	134	0.01	<0.01	3290	74	3267	101	3060	94	3084	110	0.18	0.05
Protein	g/14184 kJ	87.7	1.0	89.3	1.5	89.2	2.0	86.6	2.5	84.0	2.3	0.18	0.052	90.4	1.5	88.6	2.0	86.1	1.9	82.9	2.2	0.04	<0.01
Vitamin A†	μg RE/4184 kJ	859	23	908	36	891	53	803	45	782	58	0.06	0.02	895	38	886	52	807	49	813	57	0.43	0.13
Vitamin B1	mg/4184 kJ	1.0	0.0	1.00	0.02	0.98	0.03	0.93	0.03	0.89	0.03	0.01	<0.01	0.99	0.02	0.98	0.02	0.93	0.02	0.91	0.03	0.02	<0.01
Vitamin B2	mg/4184 kJ	1.7	0.0	1.8	0.0	1.7	0.1	1.6	0.1	1.6	0.1	<0.01	<0.01	1.8	0.0	1.7	0.1	1.6	0.1	1.6	0.1	0.12	0.02
Niacin‡	mg NE/4184 kJ	36.9	0.5	37.6	0.7	37.8	0.9	36.4	1.1	35.1	1.2	0.11	0.053	38.0	0.7	37.4	1.0	36.3	0.9	34.7	1.1	0.07	0.01
Vitamin B6	mg/4184 kJ	1.6	0.0	1.6	0.0	1.6	0.0	1.5	0.0	1.4	0.1	<0.01	<0.01	1.6	0.0	1.6	0.0	1.5	0.0	1.4	0.0	<0.01	<0.01
Vitamin B12	μg/4184 kJ	10.4	0.3	10.8	0.4	11.5	0.6	10.3	0.7	8.8	0.6	<0.01	0.02	10.8	0.5	11.5	0.6	10.3	0.6	8.5	0.7	0.01	0.01
Folate	μg/4184 kJ	416	8	442	12	441	17	387	15	368	21	<0.01	<0.01	428	12	440	17	395	16	393	18	0.10	0.05
Vitamin C	mg/4184 kJ	132	3	144	5	140	7	121	6	110	8	<0.01	<0.01	136	5	141	7	124	6	120	7	0.10	0.04
Calcium	mg/4184 kJ	675	14	718	21	677	27	647	36	611	37	<0.01	<0.01	703	23	678	32	651	30	639	35	0.40	0.09
Magnesium	mg/4184 kJ	321	4	329	6	330	7	311	8	308	11	0.050	0.02	328	6	329	8	313	8	311	9	0.24	0.07
Iron	mg/4184 kJ	9.8	0.1	10.2	0.2	10.3	0.3	9.4	0.3	9.2	0.3	0.01	<0.01	10.0	0.2	10.3	0.3	9.4	0.3	9.4	0.3	0.08	0.04
Zinc	mg/4184 kJ	10.3	0.1	10.4	0.2	10.5	0.2	10.2	0.3	9.9	0.2	0.39	0.10	10.6	0.1	10.4	0.2	10.1	0.2	9.7	0.2	<0.01	<0.01
Copper	mg/4184 kJ	1.4	0.0	1.4	0.0	1.5	0.0	1.4	0.0	1.4	0.0	0.34	0.64	1.4	0.0	1.5	0.0	1.4	0.0	1.4	0.0	0.14	0.10

SE, Standard error

*An SMS meal is a meal that includes staple, main, and side dishes together in one meal.

†Sum of retinol, β-carotene/12, α-carotene/24, and cryptoxanthin/24

‡Sum of niacin and protein/6000

§Adjusted for confounding variables of sex (men or women), age (years, continuous), body mass index (kg/m^2, continuous), marital status (married or separated/divorced/widowed/unmarried),
skipping breakfast (yes or no), food label use (yes or no), currently smoking (yes or no), walking duration/day (>1 h / 30 min–1 h / <30 min or rare),
frequency of alcohol consumption/week (>5 days /1–4 days/<1 day or teetotal).

∣∣P-values are presented for the Kruskal—Wallis test to analyze the between-group differences in nutrient intakes.

¶Linear regression analysis was used to test the trend of nutrient intakes across the four groups.

**P-values are presented for covariate analysis to analyze the between-group differences in nutrient intakes.

Table S2. Habitual daily food group intakes (g/4184kJ) among participants (n=331) categorized into four groups by SMS meal* frequency

	Crude Model										Multivariate Model†									
	Almost every day (n = 132)		4–5 days/week (n = 65)		2–3 days/week (n = 74)		Once a week or never (n = 60)				Almost every day (n = 132)		4–5 days/week (n = 65)		2–3 days/week (n=74)		Once a week or never (n=60)			
Food group	Mean	SE	Mean	SE	Mean	SE	Mean	SE	P‡	P trend§	Mean	SE	Mean	SE	Mean	SE	Mean	SE	P∣∣	P trend§
Cereals	194.3	3.7	207.4	3.6	216.0	4.3	219.5	5.2	0.13	0.02	203.6	6.2	208.5	8.5	212.8	8.0	201.7	9.3	0.76	0.84
Rice	135.9	3.8	146.6	3.6	149.8	4.4	140.7	4.6	0.48	0.51	144.8	6.4	147.4	8.7	146.4	8.1	124.5	9.5	0.25	0.19
Bread	33.5	1.1	38.5	1.5	40.5	1.4	52.1	2.4	<0.01	<0.01	35.4	2.5	38.4	3.4	40.0	3.2	48.5	3.7	0.049	<0.01
Noodles	24.9	0.9	22.2	0.9	25.7	1.0	26.8	1.1	0.55	0.91	23.4	1.6	22.7	2.2	26.4	2.0	28.7	2.4	0.19	0.052
Pulses	40.0	1.6	36.9	1.4	31.0	1.5	32.2	1.9	0.01	<0.01	37.0	2.6	37.0	3.5	31.7	3.3	37.7	3.8	0.53	0.66
Potatoes	23.6	1.2	19.4	0.8	18.2	0.8	19.1	1.1	0.15	<0.01	22.9	1.7	19.9	2.3	18.2	2.1	20.1	2.5	0.37	0.18
Sugar and confections	28.5	1.0	27.4	1.1	28.2	1.2	33.9	1.3	0.31	0.42	27.4	1.8	28.0	2.5	28.5	2.3	35.2	2.7	0.11	0.047
Sugar	0.8	0.1	1.1	0.1	0.8	0.1	1.8	0.2	0.04	0.01	0.9	0.2	1.2	0.3	0.7	0.3	1.5	0.3	0.25	0.44
Confections	26.2	1.0	24.8	1.1	25.8	1.1	31.0	1.3	0.37	0.42	25.0	1.8	25.4	2.5	26.2	2.3	32.6	2.7	0.13	0.051
Fat and oil	6.4	0.2	6.2	0.1	6.2	0.2	6.3	0.2	0.91	0.59	6.6	0.3	6.2	0.4	6.0	0.3	6.3	0.4	0.63	0.36
Fruits	41.4	2.2	40.2	2.1	34.7	1.7	23.1	1.6	<0.01	<0.01	37.2	3.1	41.9	4.3	36.6	4.0	28.3	4.7	0.20	0.18
Total vegetables	147.0	4.1	135.0	4.0	119.2	4.1	103.7	4.0	<0.01	<0.01	135.6	6.3	138.2	8.6	123.1	8.0	120.5	9.4	0.36	0.12
Green and yellow vegetables	49.9	1.9	45.3	2.0	38.3	1.8	35.1	1.7	<0.01	<0.01	44.9	2.9	46.9	4.0	40.2	3.7	42.1	4.4	0.62	0.38
Other vegetables	76.4	2.2	71.8	2.1	64.7	2.4	55.0	2.1	<0.01	<0.01	71.2	3.5	73.1	4.8	66.1	4.5	63.3	5.2	0.45	0.16
Pickled vegetables	7.4	0.5	5.6	0.3	6.2	0.4	5.1	0.3	0.67	0.48	6.9	0.7	5.6	1.0	6.5	0.9	5.9	1.1	0.72	0.55
Mushrooms	6.8	0.3	5.8	0.3	5.1	0.2	4.9	0.3	0.02	<0.01	6.5	0.5	5.8	0.7	5.2	0.6	5.5	0.7	0.45	0.15
Seaweed	6.5	0.3	6.5	0.4	4.9	0.3	3.6	0.2	<0.01	<0.01	6.2	0.6	6.7	0.8	5.1	0.7	3.8	0.8	0.050	0.02
Beverages	448.4	12.1	448.3	11.4	432.1	14.4	467.2	17.1	0.65	0.32	444.6	20.9	442.9	28.6	438.6	26.8	473.5	31.3	0.84	0.61
Fruit and vegetable juice	21.7	2.9	25.2	2.3	20.5	1.7	26.8	2.8	0.73	0.40	21.3	4.2	25.1	5.7	20.5	5.4	27.8	6.3	0.79	0.59
Tea	179.8	9.4	179.8	8.0	139.3	9.1	137.9	9.5	0.048	0.02	172.6	14.7	181.8	20.2	141.5	18.9	148.8	22.1	0.41	0.20
Coffee	163.1	6.6	144.0	5.7	161.7	8.0	195.9	10.5	0.81	0.99	160.0	12.6	149.5	17.3	161.7	16.2	196.8	18.9	0.29	0.18
Soft drinks	17.5	1.8	23.6	2.0	29.7	2.6	47.1	5.3	<0.01	<0.01	19.5	4.9	23.6	6.8	28.8	6.3	43.9	7.4	0.07	0.01
Fish and shellfish	35.7	1.1	37.0	0.9	32.7	1.1	26.1	0.9	<0.01	<0.01	34.9	1.7	37.4	2.3	33.3	2.2	26.8	2.6	0.02	0.02
Meat	44.0	1.2	39.9	1.0	41.5	1.0	37.4	1.1	0.24	0.06	44.0	1.8	39.8	2.4	40.4	2.3	38.8	2.7	0.33	0.10
Chicken	17.4	0.8	15.1	0.5	17.2	0.6	15.0	0.7	0.31	0.44	17.5	1.2	14.9	1.6	16.7	1.5	15.5	1.8	0.58	0.45
Pork and beef	26.6	0.7	24.8	0.7	24.3	0.7	22.4	0.7	0.10	0.01	26.5	1.2	24.9	1.6	23.7	1.5	23.3	1.7	0.37	0.09
Eggs	27.2	1.0	22.6	0.7	22.3	0.8	20.0	0.8	0.08	0.01	26.9	1.5	22.6	2.0	22.0	1.9	20.9	2.2	0.08	0.02
Dairy products	78.0	3.7	61.6	2.7	68.9	4.5	56.8	3.7	0.02	<0.01	73.3	6.1	62.5	8.4	69.0	7.8	66.0	9.1	0.77	0.57

SE, Standard error

*An SMS meal is a meal that includes staple, main, and side dishes together in one meal.

†Adjusted for confounding variables of sex (men or women), age (years, continuous), body mass index (kg/m^2, continuous), marital status (married or separated/divorced/widowed/unmarried), skipping breakfast (yes or no), food label use (yes or no), currently smoking (yes or no), walking duration/day (>1 h/30 min–1 h/<30 min or rare), frequency of alcohol consumption/week (>5 days/1–4 days/<1 day or teetotal).

‡P-values are presented for the Kruskal–Wallis test to analyze the between-group difference in nutrient intakes.

§Linear regression analysis was used to test the trend of food group intakes across the four groups.

∣∣P-values are presented for the covariate analysis, which analyzed the between-group difference in food group intakes.
